# Exploratory study about the relationship between leukocytosis and mental disorders in psychiatric outpatients

**DOI:** 10.1097/MD.0000000000043873

**Published:** 2025-08-15

**Authors:** Fumi Akasaka, Kuni Akasaka, Kazutake Okada, Naohito Yamaguchi, Yasuo Haruki, Sotaro Sadahiro

**Affiliations:** a Akebono Clinic, 26-1 Kaminakasone, Ishinomaki, Miyagi, Japan; b Department of Surgery, Tokai University Hospital, Isehara, Japan; c Research Division, Saiseikai Research Institute of Health Care and Welfare, Minatoku, Tokyo, Japan; d Department of Basic Medical Science, Tokai University Hospital, Isehara, Japan.

**Keywords:** leukocytosis, psychiatric disorders, white blood cells

## Abstract

Elevated levels of inflammatory cytokines and white blood cells (WBCs) have been observed in some patients with psychiatric disorders. However, the prevalence of leukocytosis, its association with psychiatric conditions, and its relevance in psychiatric outpatient care remain underexplored. This study included psychiatric outpatients who visited Akebono Clinic and underwent blood testing between October 2022 and September 2023. From patient records, we obtained age, sex, DSM-5-TR classification, and the highest and lowest WBC counts during the observation period. Leukocytosis was defined as an absolute WBC count exceeding 10,000 cells/μL. Among the 1120 psychiatric outpatients included, 187 (16.7%) exhibited leukocytosis at least once during their visits. Ten (5.7%) of the 176 patients who underwent blood testing only once had leukocytosis. This percentage was significantly higher than that reported for the general Japanese population (*P* < .001). Leukocytosis exceeded 20% for patients with schizophrenia spectrum, bipolar and related, obsessive-compulsive and related, and personality disorders. The percentages were moderate (between 10% and 20%) for those with sleep-wake, depressive, anxiety, and neurodevelopmental disorders, and low (<10%) for patients with somatic symptom and related, trauma- and stressor-related, and neurocognitive disorders. Leukocytosis had significantly higher percentages for those with schizophrenia spectrum, bipolar and related disorders, and depressive disorders than those for neurocognitive disorders (*P* < .01, *P* < .05, and *P* < .05, respectively). Leukocytosis was observed in 20.5% of patients aged 30 to 69, 9.5% of those aged 10 to 29, and 8.7% of those aged 70 to 95, indicating significant differences in age groups. Leukocytosis was considerably more frequent in psychiatric patients than in the general population. Its prevalence was especially high, exceeding 20%, in patients with schizophrenia spectrum, bipolar and related, obsessive-compulsive and related, and personality disorders, and among middle-aged individuals.

## 1. Introduction

In psychiatric outpatient care, we routinely conduct interviews, administer psychological tests, and perform periodic blood tests to assess the general condition of patients and monitor for adverse effects from prescribed medications. Nearly a century ago, a study reported that approximately 50% of inpatients with schizophrenia had white blood cell (WBC) counts exceeding 10,000/μL in peripheral blood;^[1]^ another study determined approximately 30% of 200 psychiatric inpatients had similar WBC elevations despite the absence of infection.^[2]^ More recently, elevated levels of inflammatory cytokines such as IL-1, IL-6, TNF-α, and TGF-β have been observed in patients with schizophrenia, suggesting a strong association between inflammation and the disease’s etiology.^[3,4]^

In addition to schizophrenia, leukocytosis and an elevated neutrophil-to-lymphocyte ratio have been reported in some patients with depression,^[5–8]^ anxiety disorders,^[7,9]^ bipolar disorder^[10]^ and cognitive impairment associated with Alzheimer and Parkinson diseases.^[11]^ However, the percentage of leukocytosis, its relationship with psychiatric disorders, and its clinical relevance in psychiatric outpatient care have rarely been examined.

Therefore, in this study we retrospectively investigated the relationship between leukocytosis and psychiatric disorders, the percentage of leukocytosis, and the significance of its measurement in psychiatric outpatients.

## 2. Methods

### 2.1. Participants

The study included outpatients with psychiatric disorders who visited Akebono Clinic (Ishinomaki, Japan) between October 2022 and September 2023 and underwent blood tests. From the patient records we obtained patient’ age, sex, DSM-5-TR classification,^[12]^ and the highest and lowest WBC counts observed during their visits. Patients with comorbid psychiatric conditions were categorized based on their primary diagnosis.

Patient blood samples were obtained from a medial cutaneous vein and analyzed using an automated blood analyzer.

Leukocytosis was defined as an elevation in absolute WBC count (>10,000 cells/μL), based on the Washington Manual of Medical Therapeutics, 37^th^ Edition (2022).^[13]^

Patients with leukocytosis who had concurrent inflammatory conditions such as pneumonia, trauma, sinusitis, or rheumatism at the time of blood sampling were excluded. The final number of patients included in the study was 1120.

### 2.2. Statistical analyses

Results are expressed as means ± standard deviation for WBC counts, and medians (25%, 75%) for ages, which were not normally distributed (*P* < .05, Kolmogorov–Smirnov and Shapiro–Wilk tests). The Mann–Whitney *U* test was used for a nonparametric age analysis.

A one-sample percentage test was performed to compare the leukocytosis percentage in the patients with that of the general Japanese population.

The sample size was calculated to detect a clinical difference (6% vs 0.6%) with a power of at least 0.80 and a significance level of .05. A one-sample *t*-test was conducted to compare the WBC levels from our sample with the reference value from Japanese health checkup data. A logarithmic transformation was applied to the WBC values to achieve a normal distribution (*P* > .20, Kolmogorov–Smirnov and Shapiro–Wilk tests); transformed values were used only in the statistical analyses, whereas the original values were used for presentation.

The percentages of leukocytosis across categorical variables (diagnostic or age groups) were initially evaluated using the chi-square test with Yates continuity correction. If the chi-square test was significant, pairwise comparisons were conducted using Fisher exact test with Bonferroni correction as post hoc Dunnett comparisons.

A *P*-value < .05 (two-tailed) was considered significant. All statistical analyses were performed using JMP 3.0 software (SAS Institute, Cary).

This study was conducted following the Ethical Guidelines for Medical and Health Research Involving Human Subjects and was approved by the Japanese Association of Neuro-Psychiatric Clinics (Approval No. 2023-15). The study details were publicly disclosed and patients were informed that they could withdraw consent. However, none of the patients or their families declined participation.

## 3. Results

### 3.1. Participants

Of the 1120 patients, 673 (60.1%) were females and 447 (39.9%) were males. The median age was 52 (38, 69) years for females and 46 (35, 59) years for males. The female patients were significantly older than the male patients (*P* < .001, Mann–Whitney *U* test).

Depressive disorders were the most common diagnosis (361 patients), followed by anxiety (205 patients) and neurodevelopmental (198 patients) disorders.

### 3.2. Leukocytosis percentages

Among the total sample, 187 of 1120 patients (16.7%) exhibited leukocytosis at least once during their visits. Among the 176 patients who received a blood test only once during their visits, 10 (5.7%) had leukocytosis.

### 3.3. Leukocytosis percentages by disease according to DSM-5-TR classifications

Table 1 lists the leukocytosis percentages for 11 disorders, each had 10 or more patients with the disorder. The equality of leukocytosis percentages was tested using a chi-square test. As the percentages differed significantly among the groups (*P* = .018), post hoc Dunnett comparisons were performed using Fisher exact test (Table 1).

**Table 1:** Leukocytosis percentages by disease according to DSM-5-TR classifications.

DSM-5-TR classification	WBC >10.0	WBC <10.0	Total	*P*-value*
	n (percentage)	n (percentage)	n	
Schizophrenia	19 (27.1%)	51 (72.9%)	70	.00064**
Bipolar and related disorders	9 (23.7%)	29 (76.3%)	38	.0047***
Obsessive-compulsive and related disorders	2 (20.0%)	8 (80.0%)	10	.085
Personality disorders	3 (20.0%)	12 (80.0%)	15	.047
Sleep-wake disorders	15 (19.2%)	63 (80.8%)	78	.0099
Depressive disorders	66 (18.3%)	295 (81.7%)	361	.0043***
Anxiety disorders	33 (16.1%)	172 (83.9%)	205	.014
Neurodevelopmental disorders	30 (15.2%)	168 (84.8%)	198	.022
Somatic symptom and related disorders	2 (9.5%)	19 (90.5%)	21	.24
Trauma- and stress-related disorders	4 (6.0%)	63 (94.0%)	67	.65
Neurocognitive disorders	1 (2.3%)	43 (97.7%)	44	–
Others	3	10	13	–
Total	187 (16.7%)	933 (83.3%)	1120	–

WBC = white blood cell.

* *P*-values calculated using Fisher exact test compared to neurocognitive disorders group; *P* < .005 was considered significant.

** *P* < .01, after Bonferroni correction.

*** *P* < .05.

The percentage of leukocytosis was >20% for those with schizophrenia spectrum, and bipolar and related, obsessive-compulsive and related, and personality disorders. The leukocytosis percentages for schizophrenia (27.1%, *P* < .01), bipolar and related disorders (23.7%, *P* < .05) and depressive disorders (18.3%, *P* < .05) were significantly higher (after Bonferroni correction) compared to that for neurocognitive disorders (2.3%), which was the group with the lowest percentage.

### 3.4. Leukocytosis percentages by age

The relationship between leukocytosis and age is summarized in Table 2. For the age groups 30 to 39, 40 to 49, 50 to 59, and 60 to 69 years, the percentage was >15%. We recategorized the age ranges into 3 groups: 14 to 29 years (younger), 30 to 69 years (middle-aged), and 70 to 95 years (older). A chi-square test revealed significant differences in leukocytosis percentages between the 3 groups (*P* = .000012).

**Table 2:** Leukocytosis percentages by age groups.

a.				b.			
Age-range	WBC >10.0	WBC <10.0	Total	Age-range	WBC >10.0	WBC <10.0	Total
(year)	n (percentage)	n (percentage)	n	(year)	n (percentage)	n (percentage)	n
14–19	3 (13.0%)	20 (87.0%)	23	14–29 (younger)	16 (10.1%)	142 (89.9%)	158
20–29	13 (9.6%)	122 (90.4%)	135	30–69 (middle-aged)	152 (20.5%)	591 (79.5%)	743
30–39	35 (18.9%)	150 (81.1%)	185	70–95 (older)	19 (8.7%)	200 (91.3%)	219
40–49	46 (21.1%)	172 (78.9%)	218	Total	187 (16.7%)	933 (83.3%)	1120
50–59	46 (24.0%)	146 (76.0%)	192	–	–	–	–
60–69	25 (16.9%)	123 (83.1%)	148	–	*P*-values*	–	–
70–79	17 (12.7%)	117 (87.3%)	134	–	–	younger vs middle-aged	.0022**
80–89	2 (2.7%)	73 (97.3%)	75	–	–	Young vs older	.72
90–95	0 (0.0%)	10 (100.0%)	10	–	–	middle-aged vs older	.000032***
Total	187 (16.7%)	933 (83.3%)	1120	–	–	–	–

WBC = white blood cell.

* *P*-values were calculated using Fisher exact test for each pair among the 3 groups; *P* < .017 was considered significant.

** *P* < .01.

*** *P* < .001, after Bonferroni correction.

The percentage in the middle-aged group was significantly higher than that in the younger and the older age groups (*P* < .01 and *P* < .001 respectively, after Bonferroni correction). No significant difference was observed between the younger and the older groups.

### 3.5. Impact of lithium treatment

Lithium treatment for bipolar and related disorders can transiently increase WBC counts.^[14,15]^ We examined the presence or absence of lithium use in patients diagnosed with bipolar and related disorders.

Among the 38 patients in this category, 14 (36.8%) were treated with lithium. Leukocytosis was observed in 2 of these 14 patients (14.3%) receiving lithium and in 7 of the 24 patients (29.2%) not receiving lithium. No significant association was observed between lithium treatment and leukocytosis (*P* = .44, Fisher exact test).

## 4. Discussion

An elevated WBC count is typically a physiological response of the bone marrow to infection, inflammation, corticosteroid use, β-agonist or lithium therapy, or splenectomy.^[13]^ Leukocytosis is defined as an increase in the absolute WBC count exceeding 10,000 cells/μL.

In Japanese adults, normal WBC counts range from 3300 to 8600 cells/μL, as established by Ichihara et al in a study of 6345 healthy healthcare workers aged 18 to 65 years old, with a body mass index ≤ 27, daily ethanol consumption <75 g, cigarette smoking <20 per day, and no regular medication use. These values are used as reference ranges for health checkups in Japan.^[16]^

Takahashi et al reported that 13 of 2182 individuals (0.6%) who undergo routine health checkups have leukocytosis, defined as a WBC count above 10,000/μL.^[17]^ In comparison, among the 176 patients in the current study who received only 1 blood test, 10 (5.7%) were observed to have leukocytosis. This percentage was significantly higher than that observed in the Japanese population (0.6%), with an odds ratio of 10.0 (95% CI: 3.87–25.2; *P* < .05, Fisher exact test).

The mean WBC count for these 176 patients was 6000 ± 1300 (/μL), which was significantly higher than the mean of 5400 ± 1300 (/μL) reported for healthy Japanese individuals by Ichihara et al^[16]^ (*P* < .001, one-sample *t*-test).

A strong association between inflammatory cytokines and psychiatric disorders such as schizophrenia autism, depression, and anxiety-based disorders has been previously reported.^[18–21]^ Milhorat et al determined that among 200 psychiatric inpatients, 33.5% had WBC counts above 10,000/μL; only 22% of those patients had infectious diseases such as upper respiratory infection, chronic sinusitis, or pyelitis, the remaining 78% had no identifiable infections. These findings suggest that elevated WBC counts are considerably more common in patients with psychiatric disorders than in the general population.^[2]^ However, the causes and mechanisms are not well understood.

The current study found that the percentage of those with leukocytosis varied by psychiatric disorder and age group. Significantly higher rates were observed for patients with schizophrenia spectrum, and bipolar and related and depressive disorders compared with those with neurocognitive disorders. Given sufficient sample sizes, conditions such as obsessive-compulsive and related, personality, and sleep-wake disorders may demonstrate significantly elevated leukocytosis rates.

An association between lithium therapy and leukocytosis for patients with bipolar and related disorders was not demonstrated in the current study. Although previous research of the general population, such as a study conducted in Denmark, has reported that smoking increases WBC count,^[22]^ this relationship could not be evaluated in this study because smoking status was undocumented in a large number of patient records.

We intended to investigate other inflammatory biomarkers, such as neutrophil count and C-reactive protein levels in patients with leukocytosis: however, most patients only had information for a WBC count from routine blood tests due to the restrictions under the Japanese health insurance system.

Future prospective studies are needed to examine the relationship between longitudinal changes in WBC counts and the clinical course of psychiatric disorders. Such investigations may help determine whether WBC count can serve as a reliable biomarker in the context of psychiatric outpatient care.

### 4.1. Limitations

This study has certain limitations. It is a retrospective analysis that was conducted at a single center, which may limit the generalizability of the findings. Due to incomplete documentation, we could not analyze some potentially relevant variables such as smoking status and other inflammatory markers. Further research involving larger, multicenter populations is necessary to validate and expand upon these findings.

## Author contributions

**Conceptualization:** Fumi Akasaka, Sotaro Sadahiro.

**Data curation:** Fumi Akasaka, Kuni Akasaka.

**Formal analysis:** Kazutake Okada, Naohito Yamaguchi, Yasuo Haruki.

**Writing – original draft:** Fumi Akasaka, Sotaro Sadahiro.

**Writing – review & editing:** Yasuo Haruki, Sotaro Sadahiro.
